# Danger signals in trauma

**DOI:** 10.1007/s00068-018-0962-3

**Published:** 2018-05-04

**Authors:** Borna Relja, Katharina Mörs, Ingo Marzi

**Affiliations:** Department of Trauma, Hand and Reconstructive Surgery, University Hospital Frankfurt, Goethe University, 60590 Frankfurt, Germany

**Keywords:** DAMP, Trauma, Injury, Biomarker, Inflammation

## Abstract

This review summarizes a short list of currently discussed trauma-induced danger-associated molecular patterns (DAMP). Due to the bivalent character and often pleiotropic effects of a DAMP, it is difficult to describe its “friend or foe” role in post-traumatic inflammation and regeneration, both systemically as well locally in tissues. DAMP can be used as biomarkers to indicate or monitor disease or injury severity, but also may serve as clinically applicable parameters for better indication and timing of surgery. Due to the inflammatory processes at the local tissue level or the systemic level, the precise role of DAMP is not always clear to define. While in vitro and experimental studies allow for the detection of these biomarkers at the different levels of an organism—cellular, tissue, circulation—this is not always easily transferable to the human setting. Increased knowledge exploring the dual role of DAMP after trauma, and concentrating on their nuclear functions, transcriptional targets, release mechanisms, cellular sources, multiple functions, their interactions and potential therapeutic targeting is warranted.

## Introduction

Traumatic injury, a major contributor to worldwide mortality, is one of the world’s most relevant but neglected health concerns [1, 2]. Next to the severe injury itself, which often results in immediate or early death at the scene or within only of a few hours, in the later post-injury phase, a large number of trauma patients die due to inflammation-related post-injury complications, which affect the immune system homeostasis, ending up in e.g., sepsis, septic shock, or multiple organ dysfunction syndrome (MODS) [2–6]. For several decades, research on post-traumatic complications has assumed a biphasic post-traumatic inflammation model. This model describes an initial proinflammatory systemic inflammatory response syndrome (SIRS), which was assumed to be mainly driven by the innate immune system, and a “counterbalancing” compensatory anti-inflammatory response syndrome (CARS) [3, 5]. However, the theory of a simultaneous SIRS–CARS paradigm has been widened for further ongoing injury-caused inflammatory processes, as, for example, the organism’s effort to strike the delicate balance between a sufficient defense against putative pathogens entering through eventual wounds on the one hand, and reducing collateral damage by immune cells on the other hand [7–9]. Furthermore, next to alterations of the innate immune system, the post-traumatic immunosuppression has been closely linked to modulations of the adaptive immune system, e.g., a shift from a T-helper cell type (Th)1- to a Th2-mediated immune response [8–11]. Additionally, notably in the last two decades the biological host response to trauma, which initially has been characterized by massive cytokine release as well as the activation and recruitment of effector cells including antigen presenting cells, has further employed a large number of both microbial and host alarmins [12–17]. Taken together, on a biochemical level, the post-traumatic immune response is not only activated by foreign non-self material, but includes endogenous factors as well, so-called danger-associated molecular pattern (DAMP), which are released from necrotic or physiologically “stressed” cells, and thus can initiate as well as recruit effector cells of the immune system [18–20]. A large number of those endogenous nuclear or cytosolic triggers have been described to initiate and perpetuate the systemic post-traumatic and/or noninfectious inflammatory response; however, the knowledge on their precise role still remains unknown [21–23]. In contrast, so-called pathogen-associated molecular patterns (PAMP) are compromised of the infectious and pathogen-induced highly conserved structures, such as CpG motifs [24, 25]. The major DAMP, which are involved in endogenous signals originating from stressed, injured, or necrotic cells in the setting of trauma will be examined in this review.

## Recognition, signaling and cellular response

The immune system, which has evolved over millions of years, can not only discriminate between self and non-self, but between safe and dangerous as well, as presented by Polly Matzinger in the “Danger Model”, expanding the work of Janeway and others [18, 19, 24, 26, 27]. Thus, this complex response to stress employs numerous equivalent or comparable components of PAMP or DAMP, which can be found in most vertebrates, invertebrates and even plants [24, 27].

As very potent triggers of inflammation, PAMP and/or DAMP are sensitized and recognized via pattern recognition receptors (PRR) [28, 29]. Several classes of PRR have been identified so far, including the most prominent group of toll-like receptors (TLR), nucleotide oligomerization domain (NOD)-like receptors (NLR), members of the C-type lectin receptors (CLR) like mannose binding lectine (MBL) or receptor for advanced glycation end products (RAGE) among others [28, 30–32].

In mammalian thirteen and in human ten different TLR types have been identified so far [33–35]. Apart from their similar structures, they are either localized on the cell membrane (TLR1, 2, 4, 5 and 6), or on intracellular compartments, i.e., endosome membrane (TLR3, 7, 8 and 9) [28, 31]. Membrane-bound TLR recognize microbial components and environmental danger signals, such as lipopolysaccharide (LPS) of Gram-negative, lipoteichoic acid (LTA) or peptidoglycan of Gram-positive bacteria, or even liporabinomannan (LAM) of mycoplasma and, e.g., endogenous high-mobility group box (HMGB) proteins or HEME, which are released from distressed cells, respectively [28, 36–40]. Intracellular TLR recognize predominantly nucleic acids derived from bacteria and viruses, such as single or double-stranded RNA from viruses, unmethylated CpG motifs, or purine analogues as well as other components of cellular stress [28].

Upon their activation, PRR transduce signals intracellularly, e.g., via mitogen-activated protein kinase (MAPK) signaling pathways to nuclei, where diverse transcription factors, among others the nuclear factor ‘kappa-light-chain-enhancer’ of activated B cells (NF-κB) become activated, subsequently inducing a cellular response [41, 42]. Here, one prominent example constitutes the MyD88 pathway [43]. TLR3 is the only TLR not using the MyD88-dependent pathway for signal transduction [44]. The cellular response upon, e.g., NF-κB activation includes the expression of, e.g., cytokines or adhesion molecules to accelerate inflammation and diapedesis of the immune effector cells [42]. In a feedback loop, those inflammatory mediators themselves can induce, e.g., DAMP to potentiate inflammation [45].

NLR are sensitizing signals of cellular stress, such as adenosine triphosphate (ATP)-induced activation of P2X7 channels and the efflux of potassium ions, host cell-free nuclear deoxyribonucleic acid (DNA), reactive oxygen species (ROS) as well as bacterial peptidoglycans, crystalline material, peptide aggregates, bacterial toxins and many others [46–48]. They are part of the multiprotein complexes, which mediate the cleavage of biologically inactive precursors of, e.g., IL-1β or IL-18 into their respective bioactive forms by the activated caspases 1 or 5, and which furthermore induce a specific form of cell death called pyroptosis [47, 49–53]. Most described inflammasomes contain a NLR sensor molecule, such as NOD-, leucin-rich repeats (LRR)-, and pyrin domain-containing (NLRP), e.g., 1 or NLRP3 [47, 52, 54, 55]. However, the direct link for the NLR-mediated inflammasome activation via binding of either PAMP or DAMP is still under discussion [48, 53]. It is strongly discussed that the secretion of inflammasome-activated cytokines must be “prepared” by a priming stimulus, which is usually supposed to be mediated by a TLR, which in turn activates the NF-κB pathway, and the transcription of IL-precursors as well as inflammasome components [56, 57]. In parallel, inflammasome induction by a DAMP as, e.g., potassium influx or binding of ATP to P2X7 is assumed to set off the NLR [53, 56–61]. Furthermore, an activation via ROS has been discussed. The dsRNA-dependent protein kinase R (PKR) has been identified as a further player of the inflammasome pathway [61–63]. Additionally, NLR with a N-terminal caspase activation and recruitment domain (CARD), which can bind RIP2, a protein kinase that can activate NF-κB and MAPK pathways inducing a response, are involved in signaling [41].

Apart from the cleavage, and subsequent activation of certain cytokines, the inflammasome complex is capable of inducing cell lysis. In addition to necrosis and apoptosis, in 2001, the new concept of pyroptosis has been introduced [64]. Pyroptosis is characterized by a well-orchestrated lysis of the cell, which is initiated by the inflammasome activation and consecutive formation of caspase-1-dependent pores of 1–2 nm width [64]. In consequence, cells are prone to swelling and lysis, but also to the release of intracellular molecules, which act as DAMP, e.g., HMGB1 or ATP. Thus, pyroptosis represents an important mode of cell death in DAMP-mediated enhancing and spreading of the immune response, which not only mainly affects the cells of the myeloid lineage but also occurs in epithelial, endothelial cells and neurons.

C-type lectin receptors (CLR) bind mainly to PAMP, such as bacterial, fungal and viral carbohydrates in a calcium-dependent manner [65]. There are two groups of transmembrane CLR, like dectin-1 or dectin-2 subgroups, and a group of soluble CLR including MBL, which comprise this large family of receptors that are expressed on most cell types including macrophages and dendritic cells (DC) [65, 66]. The signaling pathways can either directly activate NF-κB, or affect signaling by TLR, triggering cellular phagocytosis, DC maturation, chemotaxis, respiratory burst, and cytokine production [66]. In 2008, Yamasaki et al. found that macrophage-inducible C-type lectin (Mincle) senses nonhomeostatic cell death, and induces thereby the production of inflammatory cytokines and chemokines to potentiate the neutrophilic infiltration of damaged tissue [67]. Hereby, a CLR activation due to a DAMP was introduced for the first time. The authors found that Mincle-expressing cells, mainly macrophages, were activated in the presence of necrotic dead of cells due to a component of small nuclear ribonucleoprotein, spliceosome-associated protein 130 (SAP130) [67]. Recently, the endogenous Mincle ligand SAP130 was confirmed as a danger signal, which can be released by damaged cells, thereby activating inflammatory responses including inflammasome activation [68].

The cell surface receptor for advanced glycation end products of proteins (RAGE), first described in 1992, belongs to the immunoglobulin superfamily. RAGE is a multiligand receptor that binds structurally diverse array of molecules, including DAMP-like HMGB1, but also S100 family members and amyloid-β proteins [69–71]. RAGE is mainly expressed in the lung, which is intriguing its contribution to the response to environmental challenge/stress [72]. Its activation plays a role in various diseases, including sepsis and cardiovascular disease among others [73–75]. In general, RAGE is predominantly involved in the recognition of endogenous molecules released in the context of infection, chronic inflammation or physiological stress [32]. It shares numerous TLR ligands and, therefore, RAGE–ligand interactions induce numerous cellular signaling pathways, which among others lead to the activation of different transcription factors such as NF-κB, activator protein (AP)-1, or signal transducers and activators of transcription (STAT-)3 [32, 76–78]. The end products include proinflammatory mediators, such as tumor necrosis factor (TNF)α, generation of nitric oxide, several adhesion molecules and RAGE itself [77, 79–82], which is in consequence upregulated at sites of ligand interaction, thereby causing a continuous inflammatory response [77].

## Danger signals in trauma

### High-mobility group box protein

HMGB1 is one of best studied DAMP. HMGB1 is a chromatin peptide that acts as a DNA chaperon, but it is involved in binding of proteins and in DNA transcription, replication, and repair as well [83, 84]. Almost all cells constitutively express HMGB1. In physiologic conditions, HMGB1 is located in the nucleus, but it is either passively released after necrotic cell death, or actively secreted by living cells under stress in response to angiogenic and inflammatory signals [85–87]. The mechanisms of HMGB1 secretion are elusive, but it has been shown that the processing via inflammasome is involved [62]. HMGB1 can signal via RAGE, TLR2 and TLR4 as well, thereby inducing an activation of NF-κB.

Plasma levels of HMGB1 were increased within 30 min after severe trauma in humans [88]. Furthermore, HMGB1 levels correlated with the injury severity, tissue hypoperfusion, early posttraumatic coagulopathy, hyperfibrinolysis, and with complement activation as well as with the systemic inflammatory response [88]. Early increase of systemic HMGB1 after trauma indicated patients who developed organ injury, such as acute lung injury or acute renal failure in the later post-traumatic course [88]. Not only that HMGB1 was indicating organ complications, increased levels of HMGB1 were found in non-survivors from trauma as compared to survivors [88]. Levy et al. have demonstrated in their in vivo experiments that TLR4 wild-type (WT) mice had lower systemic interleukin (IL)-6 and IL-10 levels after treatment with neutralizing antibodies to HMGB1 and bilateral femur fracture compared with mice treated with nonimmune control antibody [89]. Anti-HMGB1 antibody treatment decreased serum alanine aminotransferase levels, and hepatic as well as gut mucosal NF-κB DNA binding [89]. Additionally, transient elevations in systemic HMGB1 levels were observed within 1 h post-trauma [89]. The authors have demonstrated that HMGB1, more precisely the TLR4-HMGB1 pathway, constitutes an early mediator of systemic inflammation and end-organ injury after peripheral tissue injury after trauma. Recently, it has been demonstrated in vivo that HMGB1 levels significantly increased in muscle 12 h after crush injury [90]. Furthermore, anti-HMGB1 antibody reduced the cellular apoptosis in the renal cortex, which has been associated with enhanced muscle HMGB1 levels, thus indicating a positive feedback cycle [90]. In a systematic review on the role of HMGB1 danger signaling in traumatic brain injury (TBI), HMGB1 was found to be released from damaged neurons, and furthermore elevated in patient’s serum and cerebrospinal fluid (CSF) [91]. Above that, the elaborated studies have shown that HMGB1 may serve as a prognostic biomarker and therapeutic target in patients with TBI [91]. Next to the setting of trauma, it has been demonstrated before that HMGB1 plays an important role in the initiation and propagation of inflammation and organ injury under conditions of sterile inflammation which involve ischemic processes [92–94]. In patients without preexistent lung injury, mechanical ventilation was associated with increased HMGB1 levels in bronchoalveolar lavage fluid (BAL) [95]. Furthermore, HMGB1 levels during ventilator-associated pneumonia were increased compared to healthy volunteers, but they were not different as compared to mechanical ventilation alone [95]. Intratracheally or intranasally administered HMGB1 caused acute lung injury in mice, which has been reflected by enhanced acute inflammatory injury to the lungs, neutrophil accumulation, development of lung edema, and increased pulmonary production of IL-1β, TNFα, and macrophage-inflammatory protein (MIP)-2 [96, 97]. In summary, extracellular HMGB1 coordinates numerous cellular functions, including migration, chemotaxis, activation, maturation and proliferation but also the redox status of its target cells [98–101]. However, the binding of HMGB1 to its receptors can be potentiated by binding to a whole variety of other factors, including PAMP and cytokines [101]. Table 1 provides a brief summary of only few DAMP including HMGB1 with chosen references.

**Table 1:** Brief summary of danger-associated molecular patterns (DAMP) with selected references

DAMP	Experimental data	Clinical data
HMGB1	62	86, 88, 91, 95
IL-1α	108, 109, 110, 112, 113, 114, 115, 119, 120, 121	105, 116, 117, 118
IL-1β	120	104, 116, 117, 118
IL-33	124, 126, 127, 128, 129, 130, 131, 132, 133, 134, 139, 142, 143, 144	125, 136, 137, 138, 140, 141
S100B	151, 152, 153, 154, 161	150, 155, 156, 160, 162, 163, 164, 166, 168, 169
Histones	176, 178, 180, 181, 182, 183	184
HSP	188, 189, 190, 197, 198, 199	194, 195, 196, 200, 201, 202, 203, 205
HSP27		200, 201
HSP60		194, 201, 202
HSP70	188, 189, 198, 199	195, 201, 203
HSP72	190	205
mtDNA		210
ATP	214, 215	

*ATP* adenosine triphosphate, *HMGB1* high-mobility group box, *HSP* heat-shock protein, *IL* interleukin, *mtDNA* mitochondrial deoxyribonucleic acid

### Interleukin-1

Cytokines are small messenger molecules, which are also produced, activated and released upon trauma [102]. Members of the IL-1 family IL-1α and IL-1β were the first cytokines to be discovered in 1974 by Charles A. Dinarello [103]. Even though IL-1α and IL-1β are encoded by different genes, they can be bound by the same IL-1 receptor (IL-1R) [103]. Nonetheless, IL-1α has a higher affinity for IL1-R1, and IL-1β for the soluble IL-1R2 [58]. Interleukin-1 can initiate many important immunological responses such as fever, prostaglandin synthesis, mobilization of neutrophils into tissues, activation of B- and T-cell lymphocytes, fibroblast proliferation as well as the production of antibodies, collagen and cytokines [58, 104]. In contrast to IL-1β, IL-1α is constitutively expressed mainly in resting nonhematopoietic cells, which line the gastrointestinal tract, liver, kidney and skin, but it can also be expressed in most cells and, furthermore, can be biologically active in its full-length form without its previous processing through inflammasomes, as it is mandatory for IL-1β activity [104–106]. Members of the IL-1 family also induce similar signaling cascades in their target cells via MAPK or NF-κB pathways [107]. IL-1α constitutes a dual function protein, on the one hand with being a proinflammatory activator of transcription as chromatin-associated protein, and cytokine on the other hand [108–110]. The latter is exerting its function as membrane-bound form or after being released from apoptotic or necrotic cells, thereby alerting the immune system to tissue damage [111]. The release of IL-1α into the extracellular space in stimulated cells occurs after processing of the membrane-bound IL-1α by the membrane-bound protease calpain, which is a calcium-dependent cysteine protease [112–115].

**Fig. 1:**
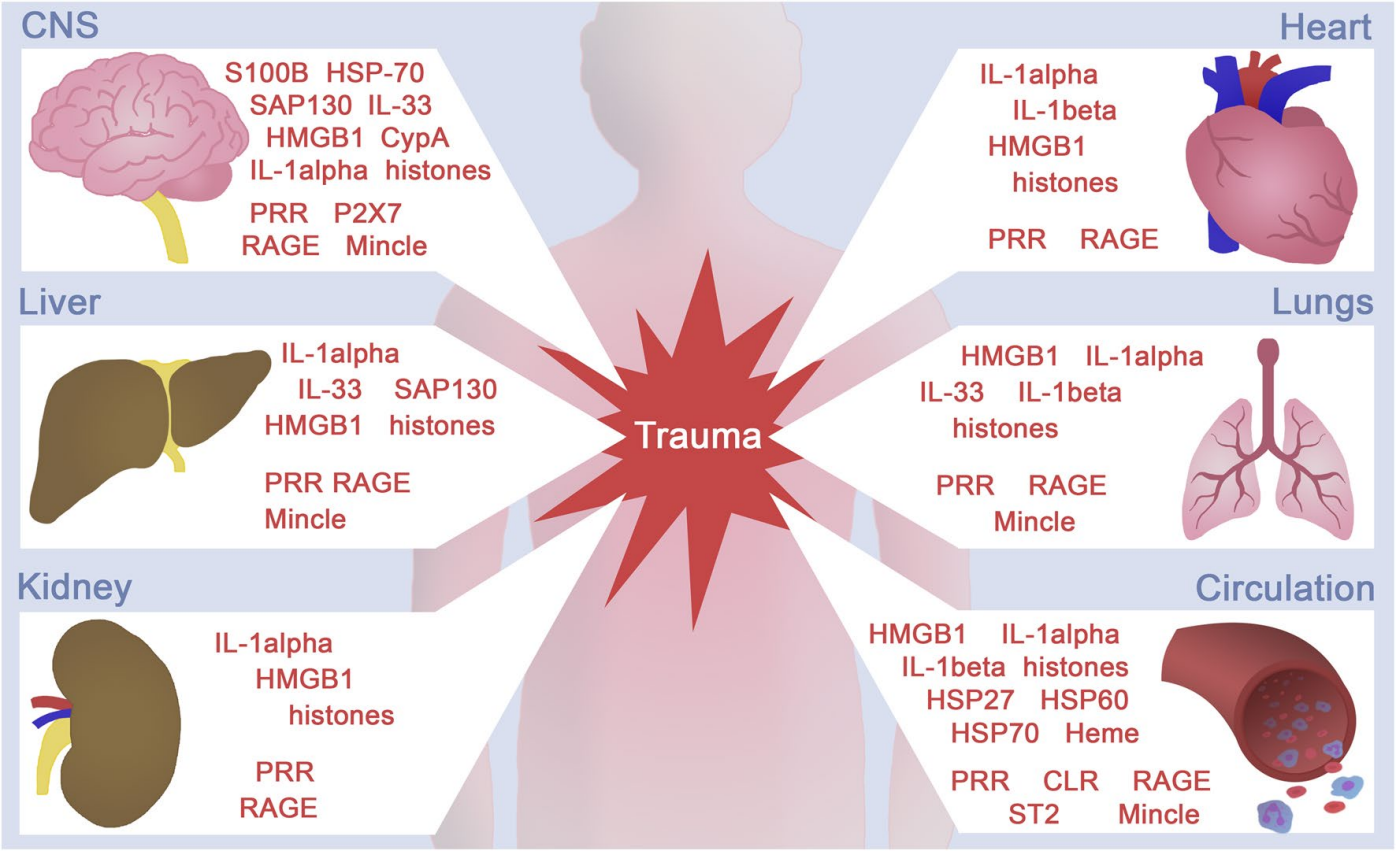
Brief concept of selected danger-associated molecular pattern (DAMP) and pattern recognition receptor (PRR) localization and their release/activation upon trauma. Trauma with tissue injury leads correspondingly to tissue- or cell-specific death, and subsequent DAMP release. *CLR* C-type lectin receptors, *CNS* central nervous system, *CypA* cyclophilin A, *HMGB1* high-mobility group box, *HSP* heat-shock protein, *IL* interleukin, *Mincle* macrophage-inducible C-type lectin, *P2X7* P2X purinoceptor 7, *RAGE* receptor for advanced glycation end products, *SAP130* spliceosome-associated protein 130, *ST2* orphan receptor ST2

There are only few studies on IL-1α in terms of trauma. Notably clinical studies with trauma patients are sparse, because most studies focussed on the role of the more prominent IL-1β. Jackman et al. tracked the plasma levels of 41 immunomodulatory proteins in 56 trauma patients beginning after trauma with a 1-year follow-up [116]. Thirty-one proteins had significant changes over time [116]. The authors have observed a mixed early response with elevated levels of IL-6, IL-10, IL-1Ra, macrophage migration inhibitory factor (MIF), myeloperoxidase (MPO), monocyte chemotactic protein-1 (MCP-1), MMP-9, and sFasL, but also simultaneously decreased levels of fractalkine, epidermal growth factor (EGF), IL-7, IL-9, IL-17, tumor necrosis factor-beta (TNFβ), MIP-1α, and macrophage-derived chemokine (MDC) and notably IL-1α [116]. In vivo data from inflammation analyses in lung tissue following blunt chest trauma by DNA microarrays have confirmed the activation of a highly complex transcriptional program in response to trauma [117]. However, regarding IL-1α, the authors represent elevated expression levels, which are concomitant with increased levels of other inflammatory and coagulatory proteins, including TNFα receptor, IL-1β, C3, NF-κB and plasminogen activator [117]. Interestingly, increased levels of IL-1 have been described to be involved in the pathogenesis of adult respiratory distress syndrome (ARDS), and subsequent idiopathic pulmonary fibrosis, sarcoidosis, as well as in certain inflammatory diseases [115, 118]. In vitro, alveolar macrophages (AM) from patients with ARDS released significantly more total IL-1 and IL-1β than controls [118]. Similar results were observed after stimulation of these cells with LPS, which indicate that AM from patients with ARDS are capable of releasing significantly more IL-1, which on the other hand may be related to the progression of acute lung injury [118]. Due to its expression pattern, IL-1α seems to play an important role in inflammation caused by necrosis or tissue damage after ischemia or hypoxia due to poor oxygen supply [115]. Under hypoxic conditions in epithelial cells, IL-1α transcription was upregulated in a process which was mediated and promoted by hypoxia-induced factor (HIF) factors [119]. During brain ischemia in a mouse model, activated platelets of the brain vasculature expressed and released IL-1α but not IL-1β, and thereby stimulated endothelial cells to secrete the chemokine CXCL1 and express cell adhesion proteins VCAM-1 and ICAM-1, thus enabling the transendothelial migration of neutrophils [120]. The authors have suggested that the activation of brain endothelium via IL-1α deriving from platelets may hide the critical step for the entry of white blood cells, which are major contributors to inflammation-mediated brain injury [120]. Similar findings regarding the role of IL-1α were reported in terms of necrotic myocytes in ischemic heart following myocardial infarction [121]. Here, IL-1α-stimulated proinflammatory cytokine expression in necrotic myocytes has been reported (IL-1β, TNFα, and IL-6) [121]. In summary, little is known about the role of IL-1α in the regulation of the post-injury inflammatory response. However, in certain settings, IL-1α constitutes the initiator of proinflammatory mechanisms that often drives chronic inflammatory diseases as well as cancer [115]. Uncovering the specific role of IL-1α in trauma and trauma-related pathologies may be promising with regard to therapies due to the availability of clinically used drugs such as anakinra, which may allow specific targeting of IL-1α in pathological conditions. Figure 1 presents some selected trauma-induced DAMP with their tissue origin.

### Interleukin-33

Interleukin-33 as the youngest member of the IL-1 family is mainly expressed in the cells lining the surfaces, which are in contact with the environment including stromal cells, such as endothelial cells, fibroblasts and epithelial cells [115, 122]. It was first described as nuclear factor from high endothelial venules (NF-HEV) [123]. As a dual-function protein, IL-33 is similar to IL-1α, and can be released upon the loss of cell integrity [124, 125]. IL-33 binds to the orphan receptor ST2 of the TLR/IL1R superfamily of receptors (also known as IL-1RL1), thereby initiating the potential signaling pathway via canonical NF-κB and MyD88 [126, 127]. Also comparable to IL-1α, IL-33 neither requires processing to maintain its activity [128]. Yet, IL-33 can be cleaved to a mature and more proinflammatory form [129]. Murine IL-33 has been cleaved by neutrophil cathepsin G and elastase, and both full-length as well as cleaved endogenous IL-33 proteins could be detected in the bronchoalveolar lavage fluid in an in vivo model of acute lung injury [129]. Thus, the relevance of IL-33 processing in a pathological environment is still not clear, but it is evident that an inflammatory microenvironment can exacerbate disease-associated functions of IL-33 via generating more active mature form [129]. Interestingly, unlike HMGB1 and IL-1α, nuclear IL-33 represses gene expression, and nuclear localization of IL-33 alters the subcellular localization of IL-33, thereby regulating the extracellular functions of IL-33 and affecting immune homeostasis [130, 131]. Furthermore, comparable to other alarmins and some members of the IL-1 family, post-translational modification of IL-33 via its oxidation (cysteine oxidation changes) can regulate its activity, and thereby restrict its inflammatory function to the local environment [115, 132]. Next to its influence on neutrophilic infiltration into tissues, biological functions of IL-33 include the modulation of the type 2 immune response, mainly Th2 cells, mast cells, eosinophils, basophils and group 2 innate lymphoid cells (ILC-2) [128, 133, 134].

With regard to trauma, there are only sparse studies, which allow to draw conclusion on the relevance of IL-33 in the regulation of the post-injury inflammatory response. Depending on the context of certain infections, IL-33 was either protective or even deleterious because it can either play a beneficial role in the resolution of inflammatory processes, or it can contribute to aggravating inflammation, respectively [115, 125, 135]. Sepsis is a systemic inflammatory condition due to bacterial infection with limited therapeutic but also diagnostic options, which frequently occurs after trauma, and hides a high risk for mortality [4, 136–138]. Interleukin-33 reduces mortality in septic mice after cecal ligation and puncture (CLP) due to increased neutrophil influx into the peritoneal cavity and enhanced bacterial clearance as compared with untreated mice [139]. Interestingly, IL-33 reduced systemic but not local proinflammatory responses by reducing the concentration of blood and lung cytokines, and thereby limiting the systemic inflammation induced by sepsis [139]. It has been shown that IL-33 prevented the downregulation of CXCR2, which is crucial for the recruitment of neutrophils from the circulation to the site of infection, thereby inhibiting their chemotaxis [139]. In summary, the authors suggested a beneficial and therapeutic potential of IL-33 in sepsis [139]. On the other hand, in certain terms of chronic inflammation, IL-33 was deleterious during the development of, e.g., asthma, or in the context of chronic obstructive pulmonary disease (COPD) and arthritic joint inflammation [140–142]. Thus, we can only hypothesize about the role of IL-33 in trauma. Gadani et al. have shown that IL-33 was highly expressed in white matter in the CNS, where it colocalized with oligodendrocyte markers as well as with astrocyte markers in gray matter [143]. The authors have demonstrated that IL-33 was released immediately after CNS injury from damaged oligodendrocytes, acting on local astrocytes and microglia to induce chemokines critical for monocyte recruitment [143]. Furthermore, mice lacking IL-33 had impaired recovery after CNS injury, which is associated with reduced myeloid cell infiltrates and decreased induction of M2 genes at the injury site [143]. Other in vivo experiments have shown that after traumatic spinal cord injury (SCI) administration of recombinant IL-33 turned beneficial [144]. The secondary damage was strongly alleviated by IL-33 by significantly decreasing tissue loss, demyelination and astrogliosis in the contused mouse spinal cord, finally resulting in dramatically improved functional recovery [144]. Similar to the above-discussed systemic and local differences in its mode of action, the authors found that IL-33 exerted effects on both, central and peripheral post-traumatic mechanisms. In spinal cord, IL-33 reduced the expression of proinflammatory TNFα, promoted the activation of anti-inflammatory arginase-1-positive M2 microglia/macrophages, and showed a tendency towards reduced T-cell infiltration [144]. In periphery, IL-33 resulted in a shift towards Th2 type cytokine profile, reduced cytotoxic TNFα-expressing CD4 cells, and increased the expression of FoxP3 T-regulatory cell marker in the spleen [144]. Taken together, these findings indicate at IL-33 to drive chemokines that recruit monocytes and polarize macrophages toward an M2 phenotype, thereby potentially protecting neurons from further damage and promoting recovery after CNS injury [145].

### S100

The family of calcium-binding homodimeric S100 proteins, or calgranulins, consists of 25 members with a large variety of intracellular and/or extracellular functions [115]. First members were isolated from bovine brains more than 50 years ago [146]. They are mainly expressed in cells of myeloid origin, predominantly in neutrophils and induced in several cell types which mediate inflammatory responses and recruit inflammatory cells to sites of tissue damage [147]. These small molecules are localized intracellularly in the cytoplasm, and here they interact with various effector molecules to regulate cell proliferation, differentiation, migration, energy metabolism, scavenging of ROS and nitric oxide, Ca^2+^ homeostasis, inflammation, apoptosis, transcriptional regulation as well as DNA repair and others [115, 148]. Certain members of the S100, such as S100A8, S100A9, and S100A12 family have been found extracellularly at high concentrations in inflamed tissue, where they exert their proinflammatory effects via binding to RAGE, TLR4 or upon interaction with other receptors [147, 149]. Importantly, not only proinflammatory functions have been described for S100 proteins, as in case of S100A8 and S100A9, some anti-inflammatory properties have been reported as well [150, 151]. The signaling pathways include the activation of kinases such as p38 MAPK, ERK1/2 and again transcription factors like NF-κB [32, 152, 153]. S100 proteins are released either passively or actively upon cell activation into the extracellular space, and act as alarmins via their interaction with different receptors to orchestrate innate and adaptive immune responses [115, 154]. Levels of S100 proteins, which have been detected in serum, urine, sputum, CSF and feces are associated with disease progression, and even more, they can be applied as biomarkers for certain pathologies, including cancer, atherosclerosis and stroke [155–160]. Several studies have reported an increase in S100A8/A9 levels in sepsis [161–163].

In the setting of trauma, S100 proteins are either released passively following cell death and tissue damage or actively to act as DAMP. Several studies have demonstrated that the level of S100B increases in CSF and plasma after injury, and even more that the increasing levels of S100B were negatively correlated to outcome from human TBI [164–166]. Nonetheless, it has been demonstrated that S100B can counteract and reduce some negative cellular consequences of injury as well [154, 165, 167]. More recent studies imply the negative role of S100B in TBI, and represent that S100B in CSF, as an astrocytic protein specific to the central nervous system is a useful marker in outcome prediction after TBI, with increased levels indicating mortality [168, 169]. Currently, S100B protein can be considered as a marker for blood–brain barrier damage, which plays an important role in the development and recovery of normal CNS after injury [170]. In terms of other S100 proteins and nervous injury, following peripheral nerve injury, an immediate acute immune response occured distally and proximally to the lesion site, and was associated with the rapid transcriptional activation of the S100a8 and S100a9 genes resulting in S100A8/A9 hetero- and homodimers [171]. These stimulated the release of chemokines and cytokines by activated Schwann cells on the other hand, and induced thereby the initial chemotactic gradient, which is responsible for the transmigration of hematogenous immune cells toward the trauma site into the injured nerve [171].

Though large number of studies deals with neurological trauma, there is some evidence that S100B is also elevated in patients with major extracranial trauma [172–174]. Anderson et al. [173] reported that S100B concentrations on admission correlate positively with greater injury severity and decreased survival in major trauma patients, independently of the presence of a head injury [174]. However, it cannot be ruled out that increased S100B after soft tissue trauma might be due to peripheral nerve injury. This study has been so far confirmed by only one further study. Here, the authors reported that circulating levels of S100B in severely injured trauma patients were increased compared to healthy volunteers [172]. Additionally, a comparison with mildly injured trauma patients has shown that S100B concentrations were associated with injury severity [172]. S100B levels correlated with systemic levels of sE-selectin, and Von Willebrand factor (vWF), which are indicating endothelial cell injury [172, 175]. Additional mechanistical analyses represented that transfection of human endothelial cells with pcDNA3.1-S100B resulted in increased apoptosis and enhanced levels of proinflammatory IL-6 and IL-8 [172]. This study has shown that S100B levels not only indicate trauma severity but also correlate with endothelial damage indicating important pathomechanical influence of S100B in the recovery from trauma as well.

### Histones

Histones are nucleoproteins that enable DNA compaction into nucleosomes. As they localize in the nucleus, their release depends on cellular necrosis and nuclear destruction. Their extracellular activity has a long history, while more than 50 years ago the first observations uncovered the antibacterial activity of these molecules [176]. It has been demonstrated that an arginine-rich fraction of calf thymus histones can kill microorganisms from diverse bacteria species including *Escherichia, Salmonella* and *Micrococcus* [176, 177]. More recently, extracellular histones, as major nuclear proteins, have been recognized as DAMP, which are involved in the pathogenesis of several diseases [178, 179]. Since the findings of Xu et al. that have demonstrated that extracellular histones released in response to inflammatory challenge contribute to endothelial dysfunction, organ failure and death during sepsis, the mechanisms of histone-mediated injury in certain organs have been extensively studied [180]. Furthermore, pharmacological targeting by antibodies to histones or by activated protein C (APC) reduced the mortality of mice in LPS, TNF or CLP models of sepsis [180]. Pathomechanically, histone administration resulted in neutrophil margination, vacuolated endothelium, intra-alveolar hemorrhage and macro and microvascular thrombosis in vivo [180].

Thus, the authors proposed extracellular histones as potential molecular targets for therapeutics for sepsis and other inflammatory diseases. Recent findings have shown that histones mainly bind and activate TLR, e.g., TLR2, TLR4 or TLR9 on various cells, similar to other DAMP, subsequently triggering inflammatory response [178, 181, 182]. Therefore, the role of circulating histones for multiple organ failure, which frequently occurs in traumatized patients, has been elaborated. C57BL/6 mice were subjected to various doses of histones and chronological evaluation of the morphological and functional changes in various organs including lungs, liver, and kidneys were performed [183]. Histone administration led to death after a dose-dependent aggravation of multiple organ injury [183]. Pulmonary and hepatic damage were evident already within 15 min, while renal injuries occurred later phase [183]. The causative effects for organ injuries were histone-driven endothelial damage, as well as the release of another DAMP, the HMGB1 [183]. The authors concluded that extracellular histones induce multiple organ injury in two progressive stages, on the one hand via direct injury to endothelial cells, and the subsequent release of other DAMP on the other hand [183]. Finally, they proposed that a therapeutic blockade of histone and HMGB1 may present a new strategy for treating histone-induced multiple organ injury.

In trauma patients it has been shown that serum histone levels were significantly increased after severe non-thoracic blunt trauma [184]. Furthermore, enhanced histone levels positively correlated with severe complications such as the incidence of acute lung injury, and dismal prognosis, as well as with markers of endothelial damage and coagulation activation [184]. Complementary in vitro studies have shown that histones directly damaged endothelial cells, stimulated cytokine release (e.g., TNFα, IL-6, and IL-10), and induced neutrophil extracellular trap formation as well as myeloperoxidase release [184]. In vivo data confirmed the significant increase of both, cytokines and markers for endothelial damage and coagulation activation immediately after trauma or histone infusion [184]. Pathological examinations showed that lungs were the predominantly affected organ with edema, hemorrhage, microvascular thrombosis and neutrophil congestion [184]. An anti-histone antibody reduced these changes and protected mice from histone-induced lethality [184]. In summary, these findings suggest that histone release plays an important pathological role in trauma-associated injuries [184].

### Heat-shock proteins

Heat-shock proteins (HSP) constitute a group of proteins that primarily act as molecular chaperones in the cytosol. They were discovered because of their specific elevated expression during the heat-shock response, which stands for their essential role in protecting cells from stress, and preparing them to survive under environmental challenges [185]. However, HSP are induced by a variety of cellular stress factors, including hypo- and hyperthermia, UV radiation, pathogens, and other forms of stress [148, 185, 186]. Its family members are named according to their molecular mass [187]. It has been under discussion, if HSP in general constitute DAMP, or if HSP70 is rather an exception. This is not as easy to evaluate as it appears, because very often contamination with molecules such as LPS or DNA can generate false-positive responses. After release, HSP70 can stimulate monocytes/macrophages, microglia and dendritic cells via the TLR2 and 4 and CD14 pathways, subsequently leading to the activation of intracellular signaling pathways [187]. Next to TLR, HSP70 binds to CD36, CD40, CD91, siglec-5 and siglec-14, lectin-like oxidized low-density lipoprotein receptor 1, and scavenger receptor class A to induce pro- or anti-inflammatory responses on a range of cells, mainly those of the innate immune system with exception of T lymphocytes [188–190]. In studies of cerebral ischemia, neurodegenerative diseases, and epilepsy, it has been shown that HSP70 reduces protein aggregates, intracellular inclusions, and apoptosis leading to neurological improvement [187, 191–193]. Regarding infectious septic complications, expression levels of HSP27, HSP60, HSP70 and HSP90 were increased in patients [194–196]. With this regard, in vivo studies have demonstrated conflictive results. Significant improvement in mortality, lung function (attenuated capillary leak), and local and systemic inflammation (prevention of the increase of MCP-1 and TNFα) in a mouse model of severe sepsis-induced ALI after therapeutical HSP90 inhibition were observed [197]. On the other hand, in CLP-induced sepsis model in HSP70.1/3 knockout mice NF-κB binding/activation, TNFα and IL-6 in lung tissue as well as mortality were increased after sepsis [198]. These data reveal that HSP70 alone confers protection from ARDS via acting at least partly through the NF-κB pathway, and thereby reducing the proinflammatory cytokine response [198]. Similar findings were reported by others; however, in their study the authors found that HSP70 may play a protective role in an age-dependent response to sepsis by preventing excessive gut apoptosis and both pulmonary and systemic inflammation [199].

Severe trauma causes enhanced expression of HSP in polymorphonuclear leukocytes during the acute post-injury phase [200]. In comparison with healthy volunteers increased expressions of HSP27, HSP70, and HSP90 in polymorphonuclear leukocytes from trauma patients were found, indicating that this enhanced expression of HSP may regulate PMNL functions [200]. Similar findings were reported for burn trauma patients, where increased expressions of HSP27, HSP60, and HSP70 in polymorphonuclear leukocytes were significantly higher than those in polymorphonuclear leukocytes from healthy volunteers [201]. Concomitant with these changes was an increase in oxidative activity in polymorphonuclear leukocytes, and markedly inhibited cell apoptosis after thermal injury [201]. Serum levels of HSP60 detected within 30 min after trauma correlated with the development of ALI after trauma [202]. In vitro, HSP60 caused the release of NO by macrophages [202]. The authors suggest that the extracellular release of the immature HSP60 may be associated with traumatic cell necrosis, and could be involved in the release of NO by immune-competent cells, subsequently inducing an activation of the local inflammatory response [202]. Increased levels of systemic HSP70 at 1–6 h after injury were found in trauma patients, and the magnitude of the increase was related to injury severity and prediction of secondary infection [203]. If HSP70 levels decreased in the period from 60 to 90 h after trauma the patient had a better outcome as compared to those patients without decreased HSP70 levels [203]. An increase in HSP70 levels between the 24–48-h period and the 60–90-h period suggested infection [203]. As suggested by others, these data support the hypothesis that HSP70 is produced as a danger signal to stimulate the immune systems of trauma patients [204]. In severely traumatized patients, systemic HSP72 levels were markedly elevated immediately after admission to the emergency department compared to healthy volunteers [205]. Moreover, elevated initial HSP72 were associated with improved survival in severely traumatized patients, although there was no difference in the overall injury severity between patients with high and low HSP72 levels [205]. Levels of HSP72 were neither related to the incidence or severity of the post-injury inflammatory response nor to organ dysfunction [205].

### Nucleic acids

Next to the DNA, and based on the endosymbiotic theory suggesting that mitochondria originate from bacteria, the cells contain mitochondrial DNA (mtDNA) as well. Nucleic acids are present in all cells, and naturally they are released into the circulation after cell necrosis and nuclear destruction, but their active release has been reported also [148, 206, 207]. This so-called extracellular DNA can be built up by either DNA or different species of RNA, and based on its origin, whether it is host or pathogen derived, is accounts for a DAMP or a PAMP, respectively [206]. Nucleic acids are recognized either by membrane-bound PRR including TLR3, TLR7, TLR8, TLR9 and RAGE, or by soluble, mainly cytosolic receptors retinoic acid-inducible gene I, melanoma differentiation-associated protein 5, and cyclic GMP–AMP [206, 208, 209].

Release of cell-free DNA plays an important role in trauma [207, 210]. The group around Zhang et al. has shown that traumatic injury causes a release of mitochondrial DAMP into the circulation, which have functionally important immune consequences [207]. These mitochondrial DAMP include formyl peptides and mtDNA as well that can activate human polymorphonuclear neutrophils via TLR [207]. The link between mitochondrial DAMP and the cellular response upon trauma is reviewed by Thurairajah et al. of this series.

### Adenosine triphosphate

Adenosine 5′ triphosphate (ATP) as another DAMP originating from mitochondria can be released extracellularly following tissue damage, and contribute to the induction of inflammation by activation and recruitment of various inflammatory cells including macrophages, neutrophils and dendritic cells [22, 211–215]. ATP signaling is transduced via purinergic receptors, e.g., by the activation of P2X7 channels and the efflux of potassium ions, with subsequent aggregation and activation of the inflammasome complex [212, 213]. Thus, proteolytical activation of pro-caspase-1 to active caspase-1 results in the processing and activation of IL-1β or IL-18 cytokine precursors as described above, thereby initiating the proinflammatory response.

### Other damp

Several other potential DAMP, which may play an important role in the initiation and regulation of the immune response, as well as in regeneration after trauma, have not been addressed in this article. For example, upon destruction of the red cells in the blood vessels, a significant quantity of hemoglobin and other contents of these cells are released into the circulation [216]. In case that this cell-free hemoglobin is not neutralized to its inert, non-toxic form by its scavenger proteins, significant damage in the vascular, perivascular and endothelial spaces occurs [216, 217].

Extracellular cyclophilin A (CypA) is a DAMP that has been associated with rheumatoid arthritis, liver injury and severe sepsis [218–220]. Cyclophilin A can act as a chemotactic agent for inflammatory cells via CD147 receptor, and directly stimulating the release of a number of inflammatory mediators. Using an in vitro shock tube model of blast TBI with SH-SY5Y human neuroblastoma cells, a potential neuroprotective mechanism involving released CypA has been suggested [221]. Accumulation of CypA in the culture medium after repeated blast exposures supported the hypothesis that extracellular CypA mediated neuroprotection [221]. These findings were confirmed by post-exposure treatment of the cells with purified recombinant CypA that resulted in a significant protection against blast-induced neuronal injury [221].

Uric acid is released from injured cells as well [222]. Uric acid is soluble inside the cells, but it precipitates to monosodium urate microcrystals in its extracellular form, exerting inflammatory properties, as evident by its accumulation in tissues and gout [21, 223]. Uric acid crystals engage the caspase-1-activating NALP3 inflammasome, resulting in the production of active IL-1β and IL-18 [224]. Macrophages from mice deficient in various components of the inflammasome complex are defective in crystal-induced IL-1β processing [224]. Furthermore, an impaired neutrophil influx is found in an in vivo model of crystal-induced peritonitis in inflammasome-deficient mice or mice deficient in the IL-1β receptor (IL-1R) [224]. Elevated levels of serum uric acid correlate with early acute kidney injury after severe burns [225]. The authors propose uric acid-related aberrant inflammation to be one of the pathogenic factors [225].

## Conclusions

This review summarizes only a short list of currently discussed DAMP that play a role in the inflammatory or regenerative response upon trauma. The list is certainly both incomplete and provides only a limited overview to the concept of trauma-induced DAMP. Due to the bivalent character and often pleotropic effects of a DAMP, it is difficult to describe its “friend or foe” role in the post-traumatic inflammation and regeneration. It is indisputable that DAMP are obligatory for the immune response upon traumatic insult, both systemically as well locally in tissues. On the one hand, they can not only be used as biomarkers to indicate or monitor disease or injury severity, but also may be clinically applicable for better indication and timing of surgery. On the other hand, they constitute either negative or positive contributing factors for the disease development. However, due to the inflammatory processes at the local tissue level or the systemic level, their precise roles are not always clear to define. While in vitro and experimental studies allow for the detection of these biomarkers at the different levels of an organism—cellular, tissue, circulation—this is not always easily transferable to the human setting. Increased knowledge exploring this dual role of DAMP after trauma, and concentrating on their nuclear functions, transcriptional targets, release mechanisms, cellular sources, multiple functions, their interactions and potential therapeutic targeting is warranted. Adjacent to in vivo studies and, furthermore, based on sometimes contradictory findings, which originate from differences between the immune system of animals and human, as discussed in this article as well, clinical research is necessary.

## Abbreviations

AM: Alveolar macrophage
APC: Activated protein C
ARDS: Acute respiratory distress syndrome
ATP: Adenosine triphosphate
AP-1: Activator protein-1
BAL: Bronchoalveolar lavage fluid
CARD: Caspase activation and recruitment domain
CARS: Compensatory anti-inflammatory response syndrome
CD: Cluster of differentiation
COPD: Chronic obstructive pulmonary disease
CLP: Cecal ligation and puncture
CLR: C-type lectin receptor
CNS: Central nervous system
CSF: Cerebrospinal fluid
CXCL: Chemokine (C-X-C motif) ligand
CypA: Cyclophilin A
DAMP: Damage-associated molecular pattern
DC: Dendritic cells
DNA: Deoxyribonucleic acid
EGF: Epidermal growth factor
GMP–AMP: Guanosine monophosphate–adenosine monophosphate
HIF: Hypoxia-induced factor
HMGB: High-mobility group box
HSP: Heat-shock protein
ICAM: Intercellular adhesion molecule
IL: Interleukin
ILC: Innate lymphoid cell
IL-1R: Interleukin-1 receptor
LAM: Liporabinomannan
LPS: Lipopolysaccharide
LRR: Leucin-rich repeats
LTA: Lipoteichoic acid
MAPK: Mitogen-activated protein kinase
MBL: Mannose-binding lectin
MCP: Monocyte chemotactic protein
MDC: Macrophage-derived chemokine
MIF: Macrophage migration inhibitory factor
Mincle: Macrophage-inducible C-type lectin
MIP: Macrophage-inflammatory protein
MMP: Matrix metalloproteinase
MODS: Multiple organ dysfunction syndrome
MPO: Myeloperoxidase
mtDNA: Mitochondrial DNA
MyD88: Myeloid differentiation primary response 88
NF-HEV: Nuclear factor from high endothelial venules
NF-κB: Nuclear factor ‘kappa-light-chain-enhancer’ of activated B cells
NLR: NOD-like receptor
NLRP: Pyrin domaine-containing nucleotide oligomerization receptor (NLR)
NOD: Nucleotide oligomerization domain
PAMP: Pathogen-associated molecular pattern
PKR: Protein kinase R
PRR: Pattern recognition receptor
RAGE: Receptor for advanced glycation end products of proteins
ROS: Reactive oxygen species
RNA: Ribonucleic acid
SAP130: Spliceosome-associated protein 130
SCI: Spinal cord injury
sFasL: Soluble Fas ligand
SIRS: Systematic inflammatory response syndrome
STAT-3: Signal transducers and activators of transcription 3
TBI: Traumatic brain injury
Th: T-helper cell
TLR: Toll-like receptor
TNF: Tumor necrosis factor
VCAM: Vascular cell adhesion molecule
VWF: Von Willebrand factor
WT: Wild type
